# Regional Cerebral Blood Flow Correlates of Neuropsychiatric Symptom Domains in Early Alzheimer’s Disease

**DOI:** 10.3390/diagnostics12051246

**Published:** 2022-05-17

**Authors:** Hyeonseok Jeong, Ilhyang Kang, Jong-Sik Park, Seung-Hee Na, Seunghee Kim, Sujung Yoon, In-Uk Song, Yong-An Chung

**Affiliations:** 1 Department of Nuclear Medicine, Incheon St. Mary’s Hospital, College of Medicine, The Catholic University of Korea, Seoul 06591, Korea; hyeonseok.s.jeong@gmail.com (H.J.); seunghee.s.kim@gmail.com (S.K.); 2 Department of Radiology, Incheon St. Mary’s Hospital, College of Medicine, The Catholic University of Korea, Seoul 06591, Korea; 3 Ewha Brain Institute, Ewha Womans University, Seoul 03760, Korea; ilhyang.h.kang@gmail.com (I.K.); sujungjyoon@ewha.ac.kr (S.Y.); 4 Department of Neurology, Incheon St. Mary’s Hospital, College of Medicine, The Catholic University of Korea, Seoul 06591, Korea; 77jjongsik@hanmail.net (J.-S.P.); seunghee.na@gmail.com (S.-H.N.); 5 Department of Brain and Cognitive Sciences, Ewha Womans University, Seoul 03760, Korea

**Keywords:** Alzheimer’s disease, neuropsychiatric symptom, regional cerebral blood flow, single-photon emission computed tomography, statistical parametric mapping

## Abstract

Although various neuropsychiatric symptoms are frequently accompanied with Alzheimer’s disease (AD) and pose a substantial burden to both patients and caregivers, their neurobiological underpinnings remain unclear. This study investigated associations between regional cerebral blood flow (rCBF) and neuropsychiatric symptom domains in early AD. A total of 59 patients with early AD underwent brain technetium-99m hexamethylpropylene amine oxime (99mTc-HMPAO) single-photon emission computed tomography (SPECT) scans. Neuropsychiatric symptoms were assessed by the Neuropsychiatric Inventory and clustered into the affective, apathy, hyperactivity, and psychotic domains. A voxel-wise multiple regression analysis was performed with four domain scores as independent variables and age, sex, and Mini-Mental State Examination scores as covariates. The affective domain score was negatively correlated with rCBF in the prefrontal cortex, thalamus, and caudate. The apathy domain score showed inverse correlations with rCBF in the prefrontal and pre/postcentral gyri and midbrain. Patients with higher hyperactivity domain scores had increased rCBF in the prefrontal and temporal lobes. The psychotic symptom domain was positively correlated with rCBF in the cuneus and negatively associated with rCBF in the prefrontal, cingulate, and occipital regions and putamen. The score of each neuropsychiatric symptom domain showed the differential correlates of brain perfusion, while altered rCBF in the prefrontal cortex was found in all domains. Although preliminary, our results may suggest common and distinct patterns of rCBF underlying neuropsychiatric symptoms in early AD. Further studies with larger samples and control participants are warranted to confirm these findings.

## 1. Introduction

Alzheimer’s disease (AD) is the leading cause of dementia and has become a cause of major medical, social, and economic burdens. Besides cognitive impairment, neuropsychiatric symptoms (NPSs) are also important characteristics of Alzheimer’s disease (AD), since one or more of these symptoms have been known to develop in more than 80% of patients during the course of AD [1]. NPSs can be shown at the earliest stage of AD, and their severity progresses exponentially over the disease course [2]. Furthermore, NPSs increase the risk of more rapid progression of dementia, functional decline in daily activities, and early death [3], and reduce the quality of life of both patients and caregivers [4]. However, clinical trials for treating NPSs in AD have reported conflicting results, and there is a considerable unmet need in clinical practice [5]. One of the reasons behind the limited therapeutic options may be that interactions between NPSs and AD pathology are not well understood. Therefore, further research on NPSs in AD is needed to provide additional insight into the underlying neurobiology of NPSs and to develop targeted early intervention strategies.

Several neuroimaging studies using various modalities have investigated the brain mechanisms of NPSs in AD [6,7,8,9]. Most of these studies have focused on the individual symptoms of NPSs in AD [10]. However, some of the NPSs, which frequently co-occur in AD patients, may share common pathophysiological processes and similar trends over time [10]. Thus, a priori-defined symptom domains may be useful to better identify the neural correlates of NPSs [7]. To date, only few studies of AD, which examined correlations between NPS domains and functional connectivity or cerebral glucose metabolism, have suggested that each NPS domain may be associated with distinct patterns of the brain functional alterations [11,12,13]. However, regional cerebral blood flow (rCBF) in relation to NPS domains remains unknown in AD patients.

This study evaluated associations between rCBF and severity of NPS domains in patients with early AD using single-photon emission computed tomography (SPECT). Based on previous neuroimaging studies [6,7,8,9], we hypothesized that each NPS domain would show distinctive spatial patterns of brain perfusion. We also hypothesized that the rCBF abnormalities of the prefrontal cortex would be shared across the domains.

## 2. Materials and Methods

### 2.1. Participants

Patients with early AD who visited the dementia outpatient clinic at Incheon St. Mary’s Hospital (Incheon, Korea) were enrolled according to the following inclusion criteria: (1) diagnosis of AD according to the National Institute of Neurological and Communicative Disorders and Stroke and the Alzheimer’s Disease and Related Disorders Association (NINCDS-ADRDA) and the Diagnostic and Statistical Manual of Mental Disorders-IV (DSM-IV) criteria; (2) a score of 0.5 in the Clinical Dementia Rating (CDR); (3) a score greater than or equal to 1 on the Neuropsychiatric Inventory (NPI); and (4) participation in brain SPECT scans. The exclusion criteria were as follows: (1) a history of stroke, head trauma, epilepsy, or cerebrovascular disease including small vessel disease based on magnetic resonance imaging (MRI); (2) a history of mixed or vascular dementia; or (3) a history of other psychiatric or neurological disorders. The study protocol was approved by the Institutional Review Board of the Incheon St. Mary’s Hospital. Informed consent was waived for this retrospective analysis.

### 2.2. Clinical Assessment

The clinical assessments, including a medical history and neurological examinations, were conducted by the neurologists. Cognitive status was assessed using the Mini-Mental State Examination (MMSE). The stages of dementia were determined using the Clinical Dementia Rating (CDR).

Twelve NPSs—delusions, hallucinations, agitation, depression, anxiety, apathy, irritability, euphoria, disinhibition, aberrant motor behavior, sleep disturbances, and eating abnormalities—were assessed using the NPI. The total score for each of the 12 symptoms, with a range of 0 to 12, is calculated by multiplying the severity scores (from 0 to 3) with the frequency scores (from 0 to 4). The scoring was based on the responses to the questions by the patient’s caregiver.

According to the previous factor analysis study on a large sample of AD patients [14], twelve NPSs were clustered into four domains as follows: (1) affective domain (anxiety and depression); (2) apathy domain (apathy and eating abnormalities); (3) hyperactivity domain (aberrant motor behavior, agitation, disinhibition, euphoria, and irritability); and (4) psychosis domain (delusions, hallucinations, and sleep disturbances). Each NPS domain score was calculated as the sum of the corresponding NPS scores [12].

### 2.3. SPECT Acquisition and Analysis

Brain SPECT scans were acquired using a dual-headed gamma camera (Discovery NM630; GE Healthcare, Milwaukee, WI, USA), which was equipped with a low-energy fan-beam collimator. After being injected with 555–740 MBq of technetium-99m hexamethylpropylene amine oxime (99mTc-HMPAO), patients were rested for 40 min before scanning. While scanning, patients were in a supine position with their eyes remaining open. Images were obtained as the camera was rotated for a total of 720°, with intervals of 6 degrees, at a rate of 12 s per frame. Reconstruction of continuous transaxial images was performed (voxel size = 1.95 × 1.95 × 2.08 mm^3^, matrix = 128 × 128 mm^2^, field of view = 250 × 250 mm^2^, 20% symmetric energy window at 140 keV). The standard ordered subset expectation maximization (OSEM) algorithm (6 iterations and 10 subsets) was performed with a Butterworth filter (cut-off frequency = 0.5 cycles/pixel, power = 10) to reduce noise.

The pre-processing and statistical analysis of all images were performed using Statistical Parametric Mapping 12 (Wellcome Centre for Human Neuroimaging, London, UK). First, the images were spatially normalized to the standard SPECT template. Then, the images were resliced with a voxel size of 2.0 × 2.0 × 2.0 mm^3^ and smoothed with a 12 mm full-width at half-maximum Gaussian kernel. Proportional scaling was applied using the cerebellum as a reference region [15]. For a voxel-wise multiple regression analysis, four NPS domain scores were entered into the model as independent variables. Age, sex, and MMSE scores were included as covariates. The voxel-level significance threshold was set at *p* < 0.005 with a cluster-level threshold of 100 voxels.

## 3. Results

A total of 59 patients (mean age = 76.5 ± 6.0 years, 17 men, education = 6.3 ± 3.8 years) with early AD (CDR stage of 0.5) were included in this study. Mean MMSE and CDR sum of boxes scores were 20.5 ± 3.6 and 2.4 ± 1.3, respectively. The frequencies and mean scores for the NPS domains and the corresponding symptoms are shown in Table 1. The hyperactivity symptom domain (67.8%) was the most frequent, followed by the affective (61.0%), apathy (57.6%), psychosis domains (32.2%).

**Figure 1:**
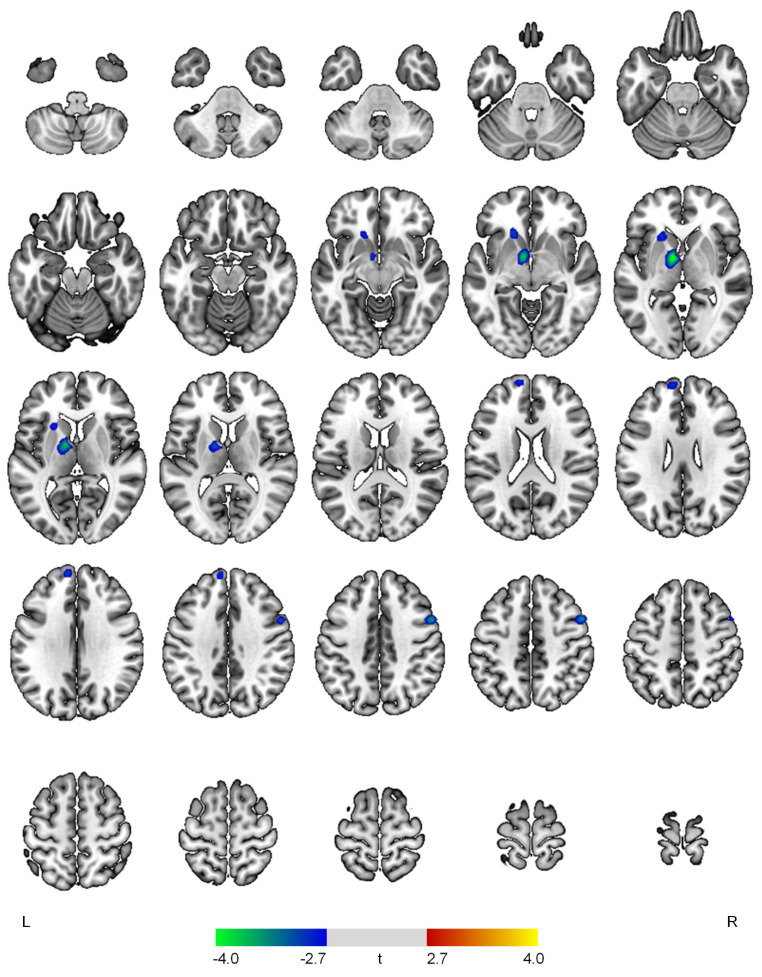
Positive (red-yellow) or negative (blue-green) correlations between regional cerebral blood flow and the affective domain in patients with early Alzheimer’s disease. The color bar represents t values at each voxel. *L*—left; *R*—right.

**Figure 2:**
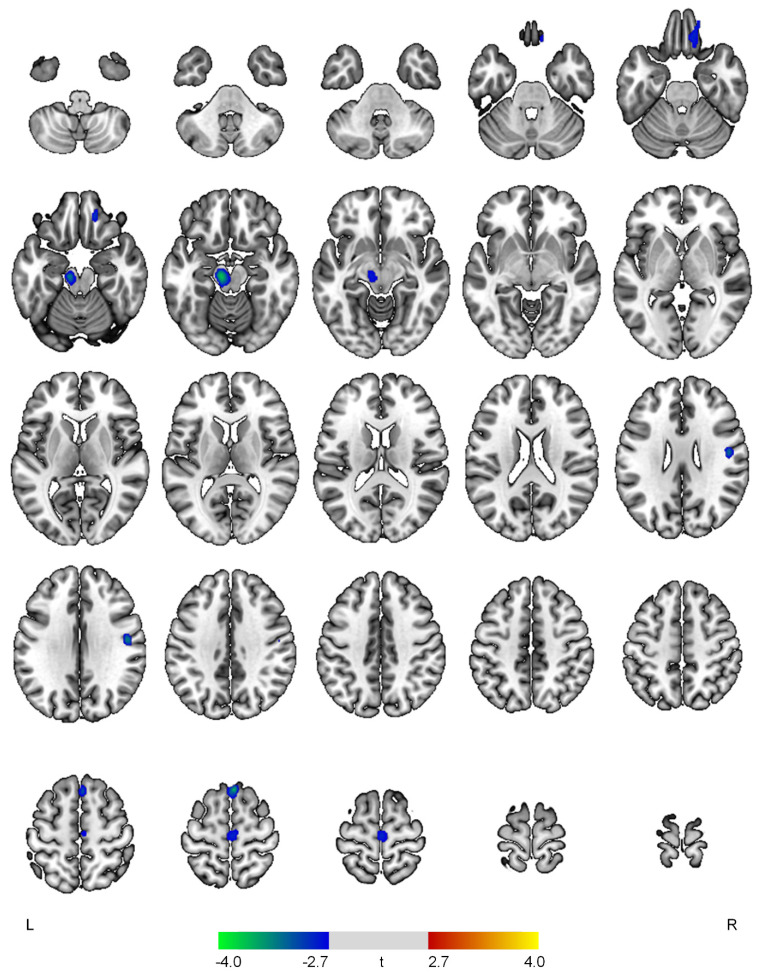
Positive (red-yellow) or negative (blue-green) correlations between regional cerebral blood flow and the apathy domain in patients with early Alzheimer’s disease. The color bar represents t values at each voxel. *L*—left; *R*—right.

**Figure 3:**
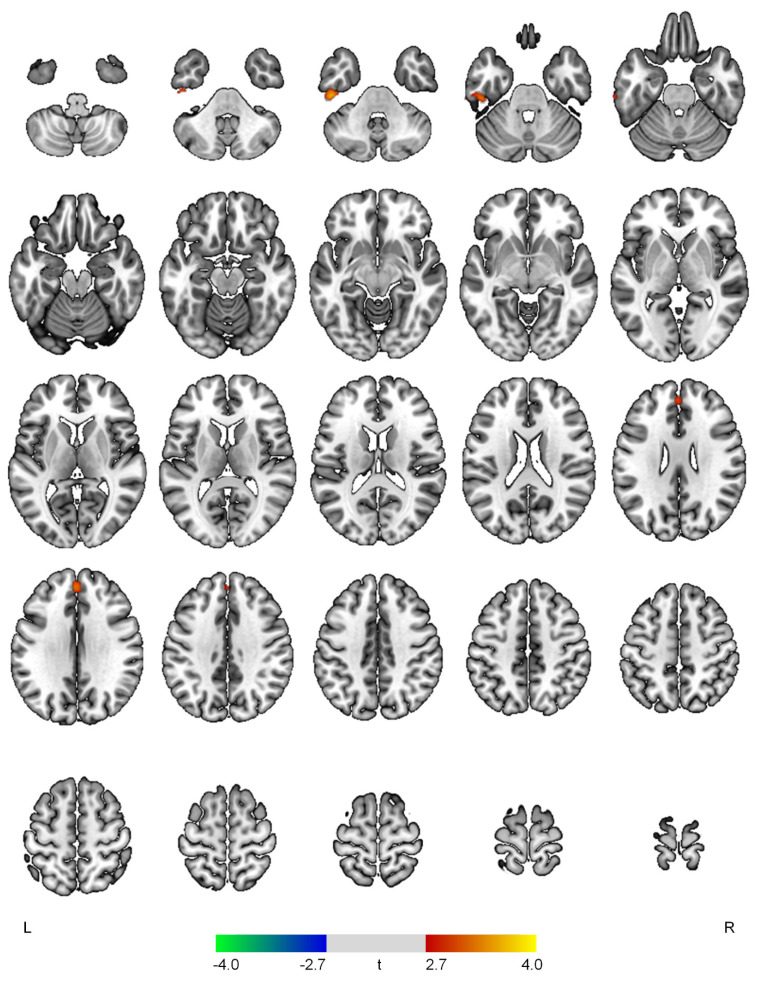
Positive (red-yellow) or negative (blue-green) correlations between regional cerebral blood flow and the hyperactivity domain in patients with early Alzheimer’s disease. The color bar represents t values at each voxel. *L*—left; *R*—right.

**Figure 4:**
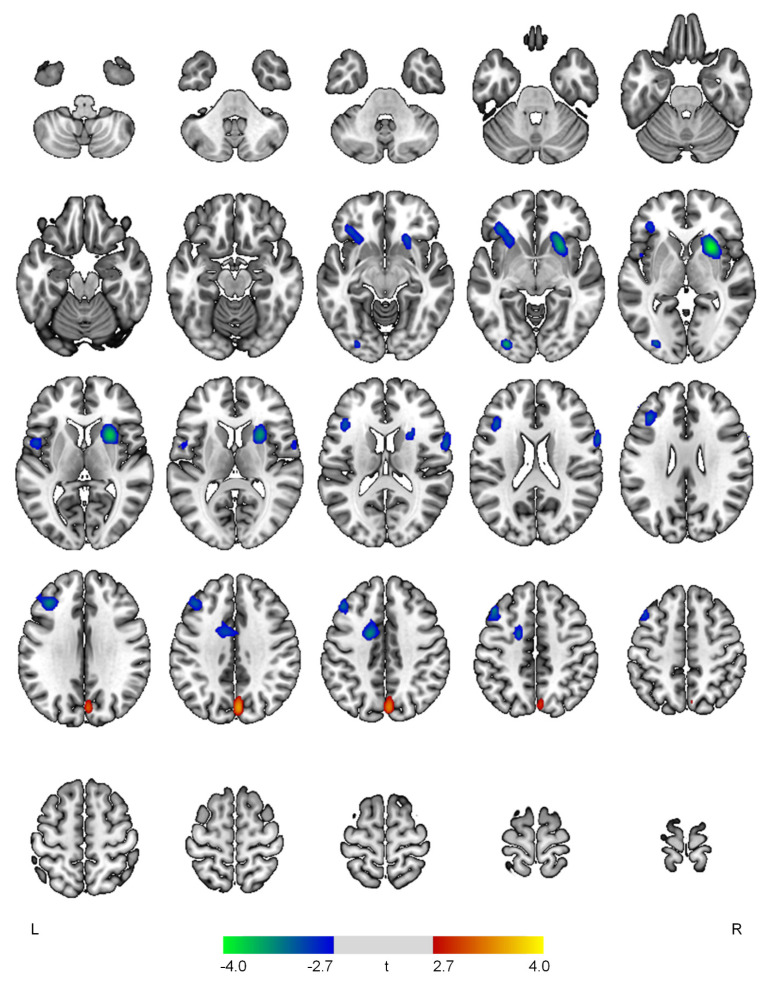
Positive (red-yellow) or negative (blue-green) correlations between regional cerebral blood flow and the psychosis domain in patients with early Alzheimer’s disease. The color bar represents t values at each voxel. *L*—left; *R*—right.

Results of the SPECT image analysis are presented in Table 2 and Figure 1, Figure 2, Figure 3 and Figure 4. The affective domain scores showed negative correlations with rCBF in the left thalamus (t = 4.08, *p* < 0.001), right precentral gyrus (t = 3.59, *p* < 0.001), left superior frontal gyrus (t = 3.06, *p* = 0.002), and left caudate (t = 3.05, *p* = 0.002) (Figure 1).

The severity of the apathy symptom domain was negatively associated with rCBF in the right superior frontal gyrus (t = 3.74, *p* < 0.001), left midbrain (t = 3.69, *p* < 0.001), right postcentral gyrus (t = 3.33, *p* = 0.001), right medial orbital gyrus (t = 3.01, *p* = 0.002), and right precentral gyrus (t = 2.89, *p* = 0.003) (Figure 2).

The hyperactivity scores demonstrated positive correlations with rCBF in the left inferior temporal gyrus (t = 3.66, *p* < 0.001) and superior frontal gyrus (t = 3.30, *p* = 0.001) (Figure 3).

The severity of the psychosis domain was positively correlated with rCBF in the right cuneus (t = 3.53, *p* < 0.001) and inversely associated with rCBF in the right putamen (t = 4.06, *p* < 0.001), left inferior occipital gyrus (t = 3.64, *p* < 0.001), left middle frontal gyrus (t = 3.49, *p* = 0.001), left anterior cingulate gyrus (t = 3.46, *p* = 0.001), left inferior frontal gyrus (t = 3.30, *p* = 0.001), right precentral gyrus (t = 3.20, *p* = 0.001), and left central operculum (t = 3.04, *p* = 0.002) (Figure 4).

## 4. Discussion

To the best of our knowledge, this is the first study to investigate associations between the NPS domains and rCBF in patients with early AD. Using the SPECT image analysis, four different NPS domains, including the affective, apathy, hyperactivity, and psychosis domains, showed distinct patterns of altered rCBF. Specifically, the affective domain was associated with decreased rCBF in the prefrontal, precentral, and subcortical regions. The apathy domain was characterized by decreased rCBF in the prefrontal, pre/postcentral, and midbrain areas. Increased rCBF in the prefrontal and temporal cortices was involved in the hyperactivity symptom domain. Lastly, altered rCBF in the prefrontal, cingulate, precentral, opercular, occipital, and subcortical regions was associated with the psychosis symptom domain. Particularly, it is noteworthy that abnormal rCBF in the prefrontal cortex was implicated in all NPS domains.

The affective domain showed negative correlations with rCBF in the superior frontal and precentral gyri, thalamus, and caudate. Previous studies have provided converging evidence that dysfunction and atrophy of the cortico-striatal-pallidal-thalamic circuits may, in part, account for the pathophysiology of major depressive disorder (MDD) [16]. This circuit consists of the prefrontal cortex, anterior cingulate cortex, basal ganglia, and thalamus and supports diverse emotional, cognitive, and motor processes. The superior frontal gyrus is considered as a core brain region for emotional processing and modulation such as rumination and cognitive control over negative feelings [17]. In AD patients with depressive or anxiety symptoms, reduced rCBF and hypometabolism of the superior frontal gyrus have been consistently reported [7,8]. The precentral gyrus is known to receive projections from the basal ganglia [18]. The abnormalities in the frontal and basal ganglia circuits may lead to psychomotor retardation and impaired action planning, which are commonly found in MDD [18]. A previous resting-state functional magnetic resonance imaging (rs-fMRI) study reported reduced regional homogeneity in both the superior frontal and precentral gyri in AD patients with depressive symptoms [19]. Deficits of the caudate may lead to psychomotor retardation and decreased hedonic drive, which are likely to be mediated by dopaminergic dysfunction [20]. A graph theoretical analysis of rs-fMRI data in AD demonstrated an inverse correlation between the affective domain scores and the closeness centrality of the caudate [13]. In addition, individuals with both AD and late-onset depression showed gray matter hypodensity in the caudate nucleus [21].

Higher apathy domain scores were associated with lower rCBF in the superior frontal, orbitofrontal, and pre/postcentral gyri and midbrain in our study. Consistent with our findings, decreased rCBF, hypometabolism, and atrophy of the superior frontal and orbitofrontal gyri have been consistently found in AD patients with apathy [6,7]. Moreover, reduced rCBF in the orbitofrontal gyrus may contribute to appetite loss in AD [22], further supporting the associations between apathy and eating abnormalities [14]. In early-onset AD, regional cerebral glucose metabolism in both the superior frontal and orbitofrontal gyri was negatively correlated with severity of the apathy domain [12]. These brain regions, which are closely involved in motivation, cognitive control, and decision making, may play important roles in apathy symptoms [23]. Although depression and apathy frequently share similar clinical manifestations, they have different neurobiological bases. While decreased rCBF in the superior frontal gyrus was found in relation to the affective domain, reduced rCBF in the orbitofrontal gyrus may be a distinct characteristic of the apathy symptom domain. AD patients with apathy demonstrated significantly lower rCBF in the postcentral gyrus and the superior frontal gyrus than those without apathy [24]. Moreover, lower functional connectivity between the planum polare and the precentral/postcentral gyri was found in apathetic patients with frontotemporal dementia and Parkinson’s disease (PD) compared to non-apathetic patients [25]. Functional deficits of the precentral and postcentral gyri in patients with apathy may be associated with a reduction in external stimuli perception and a subsequent impairment in motor response [25]. Apathy-related rCBF reduction in the midbrain has not been frequently found in previous AD research and may be one of the novel findings of this study. Increasing evidence from the preclinical and clinical studies has suggested that alterations in the midbrain structure and dopaminergic system may result in various NPSs in AD [26]. Similarly, PD patients with apathy also demonstrated lower activation of the prefrontal, striatum, amygdala, and midbrain regions in response to reward cues than those without apathy [27]. These results may underline potential links between functional deficits of the midbrain and apathy in AD patients.

In the current study, the hyperactivity symptom domain was positively correlated with rCBF in the superior frontal and inferior temporal gyri that are implicated in inhibition of impulsive thoughts and visual processing of negative emotions, respectively [28,29]. The hyperactivity symptom in AD is one of the most difficult symptoms to manage and may cause a substantial burden for the caregivers [30]. However, as compared to other symptoms in AD, the neural correlates of the hyperactivity domain in AD have remained less studied [30]. Some studies reported atrophy or hypometabolism of the prefrontal-subcortical circuit in AD patients with various hyperactivity symptoms [7,9]. Another study suggested that higher levels of agitation/aggression were associated with lower gray matter volume of the superior frontal and inferior temporal gyri [31]. However, increased glucose metabolism of the superior frontal gyrus was associated with the hyperactivity symptom domain in early-onset AD [12]. Furthermore, a recent pilot work in AD found a positive correlation between hyperactivity and cortical excitability in the dorsolateral prefrontal cortex [32]. These inconsistencies may stem from methodological issues, such as sample characteristics, evaluation methods of NPSs, and neuroimaging techniques. The implications of increased rCBF in the prefrontal and temporal regions among AD patients with hyperactivity should be further investigated.

In our study, AD patients with greater severity of psychosis symptoms showed decreased rCBF in the frontal, anterior cingulate, putamen, opercular, and inferior occipital regions and increased rCBF in the cuneus. In line with this, reduced rCBF, hypometabolism, and gray matter atrophy of the dorsolateral, inferior frontal, and anterior cingulate gyri have been consistently reported in AD patients with psychosis [6,8,13]. In addition, disruptions in the prefrontal-basal ganglia circuit and the cingulo-opercular network may lead to psychosis in various conditions [33,34]. Reduced cognitive control involved in the dorsolateral prefrontal cortex and impaired conflict monitoring related to the anterior cingulate gyrus may be implicated in the pathophysiology of schizophrenia [35,36]. In AD, delusions and hallucinations are known to be associated with extrapyramidal symptoms, suggesting the involvement of the basal ganglia circuits [37]. Cerebral atrophy of the occipital lobe was found in AD patients with visual hallucinations [38], which is the most typical form of hallucinations in AD [39]. The visual hallucinations in AD are known to be primarily caused by the ventral visual stream system including the lateral prefrontal cortex and occipital lobe [40].

The following limitations should be considered when interpreting the results of this study. First, due to the small sample size, the findings are preliminary and further larger studies are warranted to confirm the associations between NPSs and rCBF in AD. Second, as the control participants were not included in the analysis, our study demonstrated positive or negative correlations between NPSs and rCBF in the patients with both AD and NPSs. The alterations in rCBF should be compared between AD patients with NPSs and those without NPSs or healthy controls in future studies. Third, this study focused on patients with early stages of AD, considering the effects of AD pathology on cerebral perfusion. To generalize our findings, future studies including patients with various severities of AD symptoms are required. Fourth, as multiple comparison correction was not applied in the analysis, further studies with more stringent thresholds may reduce the possibility of false positives. However, a previous positron emission tomography study also used the liberal threshold of uncorrected *p* < 0.01 for the voxel-wise multiple regression analysis to examine correlations between cerebral glucose metabolism and NPS domain scores in early-onset AD [12].

## 5. Conclusions

In summary, each NPS domain score showed differential correlates of brain perfusion, while altered rCBF in the prefrontal cortex was found in all domains. Our results may suggest common and distinct patterns of rCBF which underlie NPSs in early AD. Future studies using multimodal neuroimaging techniques will further broaden our understanding of the neurobiological mechanisms underlying NPSs in AD. In addition, longitudinal studies are needed to reveal the progression of rCBF deficits and associated NPSs.

## Figures and Tables

**Table 1:** Frequencies and scores of the neuropsychiatric symptom domains and corresponding symptoms *.

Domain	Frequency (%) ^†^	Mean ± SD	Symptom	Frequency (%) ^†^	Mean ± SD
Affective	61.0	1.53 ± 1.86	Anxiety	30.5	0.61 ± 1.20
			Depression	54.2	0.92 ± 1.22
Apathy	57.6	2.54 ± 3.54	Apathy	44.1	1.07 ± 1.81
			Eating abnormalities	30.5	1.47 ± 2.81
Hyperactivity	67.8	2.71 ± 3.78	Aberrant motor behavior	17.0	0.76 ± 2.25
			Agitation	18.6	0.29 ± 0.70
			Disinhibition	23.7	0.46 ± 1.12
			Euphoria	5.1	0.07 ± 0.31
			Irritability	54.2	1.14 ± 1.57
Psychosis	32.2	1.02 ± 1.88	Delusions	11.9	0.25 ± 0.90
			Hallucinations	5.1	0.05 ± 0.22
			Sleep disturbances	25.4	0.71 ± 1.57

* Neuropsychiatric symptoms were assessed with Neuropsychiatric Inventory. ^†^ The frequency of patients with domain or symptom scores ≥ 1. *SD*—standard deviation.

**Table 2:** Brain regions with significant correlations between regional cerebral blood flow and the neuropsychiatric symptom domains.

Brain Regions	t	*p*	Cluster Size (Voxels)	MNI Coordinates
Positive correlations with the affective domain				
None				
Negative correlations with the affective domain				
L thalamus	4.08	<0.001	427	−8, −2, −2
R precentral gyrus	3.59	<0.001	172	54, 8, 42
L superior frontal gyrus	3.06	0.002	160	−8, 56, 30
L caudate	3.05	0.002	165	−18, 22, −4
Positive correlations with the apathy domain				
None				
Negative correlations with the apathy domain				
R superior frontal gyrus	3.74	<0.001	138	4, 26, 64
L midbrain	3.69	<0.001	265	−10, −20, −16
R postcentral gyrus	3.33	0.001	111	48, −10, 30
R medial orbital gyrus	3.01	0.002	177	16, 40, −22
R precentral gyrus	2.89	0.003	156	2, −20, 64
Positive correlations with the hyperactivity domain				
L inferior temporal gyrus	3.66	<0.001	315	−46, −26, −36
Superior frontal gyrus	3.30	0.001	171	0, 46, 32
Negative correlations with the hyperactivity domain				
None				
Positive correlations with the psychosis domain				
R cuneus	3.53	<0.001	305	4, −76, 34
Negative correlations with the psychosis domain				
R putamen	4.06	<0.001	921	26, 12, 0
L inferior occipital gyrus	3.64	<0.001	131	−30, −84, −4
L middle frontal gyrus	3.49	0.001	702	−44, 24, 44
L anterior cingulate gyrus	3.46	0.001	350	−14, 0, 42
L inferior frontal gyrus	3.30	0.001	368	−34, 30, −6
R precentral gyrus	3.20	0.001	191	64, 10, 20
L central operculum	3.04	0.002	118	−50, 4, 6

*L*—left; *MNI*—Montreal Neurological Institute; *R*—right.

## References

[1] Gauthier S., Cummings J., Ballard C., Brodaty H., Grossberg G., Robert P., Lyketsos C. (2010). Management of behavioral problems in Alzheimer’s disease. Int. Psychogeriatr..

[2] Fernandez-Martinez M., Molano A., Castro J., Zarranz J.J. (2010). Prevalence of neuropsychiatric symptoms in mild cognitive impairment and Alzheimer’s disease, and its relationship with cognitive impairment. Curr. Alzheimer Res..

[3] Peters M.E., Schwartz S., Han D., Rabins P.V., Steinberg M., Tschanz J.T., Lyketsos C.G. (2015). Neuropsychiatric symptoms as predictors of progression to severe Alzheimer’s dementia and death: The Cache County Dementia Progression Study. Am. J. Psychiatry.

[4] Shin I.S., Carter M., Masterman D., Fairbanks L., Cummings J.L. (2005). Neuropsychiatric symptoms and quality of life in Alzheimer disease. Am. J. Geriatr. Psychiatry.

[5] Clement A., Wiborg O., Asuni A.A. (2020). Steps Towards Developing Effective Treatments for Neuropsychiatric Disturbances in Alzheimer’s Disease: Insights From Preclinical Models, Clinical Data, and Future Directions. Front. Aging Neurosci..

[6] Rosenberg P.B., Nowrangi M.A., Lyketsos C.G. (2015). Neuropsychiatric symptoms in Alzheimer’s disease: What might be associated brain circuits?. Mol. Asp. Med..

[7] Boublay N., Schott A.M., Krolak-Salmon P. (2016). Neuroimaging correlates of neuropsychiatric symptoms in Alzheimer’s disease: A review of 20 years of research. Eur. J. Neurol..

[8] Ng K.P., Chiew H.J., Rosa-Neto P., Kandiah N., Ismail Z., Gauthier S. (2019). Brain Metabolic Dysfunction in Early Neuropsychiatric Symptoms of Dementia. Front. Pharmacol..

[9] Chen Y., Dang M., Zhang Z. (2021). Brain mechanisms underlying neuropsychiatric symptoms in Alzheimer’s disease: A systematic review of symptom-general and -specific lesion patterns. Mol. Neurodegener..

[10] Nowrangi M.A., Lyketsos C.G., Rosenberg P.B. (2015). Principles and management of neuropsychiatric symptoms in Alzheimer’s dementia. Alzheimers Res. Ther..

[11] Balthazar M.L., Pereira F.R., Lopes T.M., da Silva E.L., Coan A.C., Campos B.M., Duncan N.W., Stella F., Northoff G., Damasceno B.P. (2014). Neuropsychiatric symptoms in Alzheimer’s disease are related to functional connectivity alterations in the salience network. Hum. Brain Mapp..

[12] Ballarini T., Iaccarino L., Magnani G., Ayakta N., Miller B.L., Jagust W.J., Gorno-Tempini M.L., Rabinovici G.D., Perani D. (2016). Neuropsychiatric subsyndromes and brain metabolic network dysfunctions in early onset Alzheimer’s disease. Hum. Brain Mapp..

[13] Chang Y.T., Hsu J.L., Huang S.H., Hsu S.W., Lee C.C., Chang C.C. (2020). Functional connectome and neuropsychiatric symptom clusters of Alzheimer’s disease. J. Affect. Disord..

[14] Aalten P., Verhey F.R., Boziki M., Brugnolo A., Bullock R., Byrne E.J., Camus V., Caputo M., Collins D., De Deyn P.P. (2008). Consistency of neuropsychiatric syndromes across dementias: Results from the European Alzheimer Disease Consortium. Part II. Dement. Geriatr. Cogn. Disord..

[15] Pickut B.A., Dierckx R.A., Dobbeleir A., Audenaert K., Van Laere K., Vervaet A., De Deyn P.P. (1999). Validation of the cerebellum as a reference region for SPECT quantification in patients suffering from dementia of the Alzheimer type. Psychiatry Res..

[16] Bora E., Harrison B.J., Davey C.G., Yucel M., Pantelis C. (2012). Meta-analysis of volumetric abnormalities in cortico-striatal-pallidal-thalamic circuits in major depressive disorder. Psychol. Med..

[17] Frank D.W., Dewitt M., Hudgens-Haney M., Schaeffer D.J., Ball B.H., Schwarz N.F., Hussein A.A., Smart L.M., Sabatinelli D. (2014). Emotion regulation: Quantitative meta-analysis of functional activation and deactivation. Neurosci. Biobehav. Rev..

[18] Buyukdura J.S., McClintock S.M., Croarkin P.E. (2011). Psychomotor retardation in depression: Biological underpinnings, measurement, and treatment. Prog. Neuropsychopharmacol. Biol. Psychiatry.

[19] Guo Z., Liu X., Jia X., Hou H., Cao Y., Wei F., Li J., Chen X., Zhang Y., Shen Y. (2015). Regional Coherence Changes in Alzheimer’s Disease Patients with Depressive Symptoms: A Resting-State Functional MRI Study. J. Alzheimers Dis..

[20] Pizzagalli D.A., Holmes A.J., Dillon D.G., Goetz E.L., Birk J.L., Bogdan R., Dougherty D.D., Iosifescu D.V., Rauch S.L., Fava M. (2009). Reduced caudate and nucleus accumbens response to rewards in unmedicated individuals with major depressive disorder. Am. J. Psychiatry.

[21] Brommelhoff J.A., Spann B.M., Go J.L., Mack W.J., Gatz M. (2011). Striatal Hypodensities, Not White Matter Hypodensities on CT, Are Associated with Late-Onset Depression in Alzheimer’s Disease. J. Aging Res..

[22] Ismail Z., Herrmann N., Rothenburg L.S., Cotter A., Leibovitch F.S., Rafi-Tari S., Black S.E., Lanctot K.L. (2008). A functional neuroimaging study of appetite loss in Alzheimer’s disease. J. Neurol. Sci..

[23] Kouneiher F., Charron S., Koechlin E. (2009). Motivation and cognitive control in the human prefrontal cortex. Nat. Neurosci..

[24] Kang J.Y., Lee J.S., Kang H., Lee H.W., Kim Y.K., Jeon H.J., Chung J.K., Lee M.C., Cho M.J., Lee D.S. (2012). Regional cerebral blood flow abnormalities associated with apathy and depression in Alzheimer disease. Alzheimer Dis. Assoc. Disord..

[25] Alfano V., Longarzo M., Mele G., Esposito M., Aiello M., Salvatore M., Grossi D., Cavaliere C. (2021). Identifying a Common Functional Framework for Apathy Large-Scale Brain Network. J. Pers. Med..

[26] D’Amelio M., Puglisi-Allegra S., Mercuri N. (2018). The role of dopaminergic midbrain in Alzheimer’s disease: Translating basic science into clinical practice. Pharmacol. Res..

[27] Lawrence A.D., Goerendt I.K., Brooks D.J. (2011). Apathy blunts neural response to money in Parkinson’s disease. Soc. Neurosci..

[28] Cho S.S., Ko J.H., Pellecchia G., Van Eimeren T., Cilia R., Strafella A.P. (2010). Continuous theta burst stimulation of right dorsolateral prefrontal cortex induces changes in impulsivity level. Brain Stimul..

[29] Rosen H.J., Wilson M.R., Schauer G.F., Allison S., Gorno-Tempini M.L., Pace-Savitsky C., Kramer J.H., Levenson R.W., Weiner M., Miller B.L. (2006). Neuroanatomical correlates of impaired recognition of emotion in dementia. Neuropsychologia.

[30] Keszycki R.M., Fisher D.W., Dong H. (2019). The Hyperactivity-Impulsivity-Irritiability-Disinhibition-Aggression-Agitation Domain in Alzheimer’s Disease: Current Management and Future Directions. Front. Pharmacol..

[31] Jung M.S., Lee Y.M., Park J.M., Lee B.D., Moon E.S., Jeong H.J. (2013). Neuroanatomical Correlation of Agitation/Aggression in Alzheimer’s Disease. J. Korean Geriatr. Psychiatry.

[32] Joseph S., Zomorrodi R., Ghazala Z., Knezevic D., Blumberger D.M., Daskalakis Z.J., Mulsant B.H., Pollock B.G., Rajji T.K., Kumar S. (2020). Dorsolateral prefrontal cortex excitability assessed using TMS-EEG and its relationship with neuropsychiatric symptoms in Alzheimer’s dementia. Alzheimer’s Dement..

[33] Perez-Costas E., Melendez-Ferro M., Roberts R.C. (2010). Basal ganglia pathology in schizophrenia: Dopamine connections and anomalies. J. Neurochem..

[34] Sheffield J.M., Kandala S., Burgess G.C., Harms M.P., Barch D.M. (2016). Cingulo-opercular network efficiency mediates the association between psychotic-like experiences and cognitive ability in the general population. Biol. Psychiatry Cogn. Neurosci. Neuroimaging.

[35] Harrison B.J., Yucel M., Shaw M., Brewer W.J., Nathan P.J., Strother S.C., Olver J.S., Egan G.F., Velakoulis D., McGorry P.D. (2006). Dysfunction of dorsolateral prefrontal cortex in antipsychotic-naive schizophreniform psychosis. Psychiatry Res..

[36] Kerns J.G., Cohen J.D., MacDonald A.W., Johnson M.K., Stenger V.A., Aizenstein H., Carter C.S. (2005). Decreased conflict- and error-related activity in the anterior cingulate cortex in subjects with schizophrenia. Am. J. Psychiatry.

[37] Caligiuri M.P., Peavy G. (2000). An instrumental study of the relationship between extrapyramidal signs and psychosis in Alzheimer’s disease. J. Neuropsychiatry Clin. Neurosci..

[38] Holroyd S., Shepherd M.L., Downs J.H. (2000). Occipital atrophy is associated with visual hallucinations in Alzheimer’s disease. J. Neuropsychiatry Clin. Neurosci..

[39] Jeste D.V., Finkel S.I. (2000). Psychosis of Alzheimer’s disease and related dementias. Diagnostic criteria for a distinct syndrome. Am. J. Geriatr. Psychiatry.

[40] Collerton D., Perry E., McKeith I. (2005). Why people see things that are not there: A novel Perception and Attention Deficit model for recurrent complex visual hallucinations. Behav. Brain Sci..

